# Comparison of Morbidity Between Sentinel Lymph Node Biopsy and Elective Neck Dissection in Dogs With Head and Neck Malignancies

**DOI:** 10.1111/vco.70055

**Published:** 2026-02-17

**Authors:** Lavinia Elena Chiti, Dominique Leu, Roberta Ferrari, Ester Luconi, Patrizia Boracchi, Elisa Maria Gariboldi, Damiano Stefanello, Mirja Christine Nolff

**Affiliations:** ^1^ Clinic for Small Animals Surgery—Vetsuisse Faculty University of Zurich Zurich Switzerland; ^2^ Dipartimento di Medicina Veterinaria e Scienze Animali Università degli Studi di Milano Lodi Italy; ^3^ Dipartimento di Scienze Biomediche e Cliniche Università degli Studi di Milano Milan Italy

**Keywords:** lymph centre, mandibular node, medial retropharyngeal node, postoperative complications, staging

## Abstract

Elective neck dissection (END) and sentinel lymph node biopsy (SLNB) are suggested for nodal staging of canine head and neck malignancies (HNM). This study aims to compare the morbidity of END to SLNB. Seventy‐six client‐owned dogs with HNM that underwent END (*n* = 28) or SLNB (*n* = 48) in two institutions were retrospectively enrolled. Retrieved variables included data on signalment, lymph centre and lymph nodes, intra‐ and post‐surgical complications (PSC) of lymphadenectomy, and histopathology results. The cumulative incidence of PSC at 30 days was estimated for END and SLNB and compared with Gray's test. The influence of variables on the incidence of complications was evaluated using univariate and multivariate models. No intraoperative complication occurred. The PSC were mostly mild. Seroma was the most frequent. The cumulative incidence of PSC of lymphadenectomy at 30 days was 47.4%, and they were severe in 14% of cases. The incidence of PSC was 25% for SLNB and 85.7% for END, and the difference was statistically significant (*p* < 0.001). Clinically enlarged nodes (*p* = 0.03), institution (*p* = 0.03), increasing number of resected nodes (*p* < 0.001) and of lymph centres (*p* < 0.001) predicted a higher incidence of PSC in the univariate model. In the multivariate analysis, only the type of node management (END vs. SLNB) remained significant. Although lymphadenectomy is a well‐tolerated procedure in dogs with HNM, END was correlated with a higher risk of PSC compared to SLNB. Stratification of dogs by the risk of multiple nodal metastases is warranted to identify those who may still benefit from END despite a higher PSC risk.

## 1 Introduction

A variety of malignancies can affect the canine head and neck, and for most of them, metastatic spread to the lymph nodes has been recognised as a negative prognostic factor [1, 2, 3, 4, 5, 6, 7, 8, 9]. While physical and cytological examination of the peripheral nodes, as well as advanced diagnostic imaging techniques, are poor predictors of nodal metastases, histopathological examination of surgically excised nodes is the reported current clinical standard procedure [10, 11, 12, 13]. Due to the complex lymphatic network characterised by overlapping of drainage areas and individual variations, nodal metastases from canine head and neck tumours can be missed in 28%–62% of cases if only the ipsilateral mandibular nodes are examined [14, 15, 16, 17]. To minimise the risk of missing nodal metastases, two approaches have been translated from human to canine oncological surgery: elective neck dissection (END), which consists of a bilateral removal of multiple cervical lymph centres (the mandibular, the medial retropharyngeal, and in some papers also included the parotid lymph centre) [14, 15, 17, 18, 19]; and sentinel lymph node biopsy (SLNB), defined as the targeted excision of the first node(s) in the lymphatic basin that drain the tumour, which can be identified with various mapping techniques [20, 21, 22, 23, 24, 25, 26].

In human medicine, favourable survival rates have been reported for patients with early‐stage squamous cell carcinoma undergoing END or SLNB compared to watchful waiting [27, 28]. Similarly, recurrence‐free survival, regional lymph node metastasis‐free survival, and an overall trend towards an improved survival time were observed in patients with head and neck melanoma undergoing SLNB [29]. Potential drawbacks of END in humans include postoperative neck pain, neck fibrosis and shoulder dysfunction, which may occur in 23%–100% of patients and contribute to a reduction of the quality of life postoperatively, while SLNB has been proven to have lower morbidity compared to END, with neck‐shoulder dysfunction occurring only in 5% of patients [28, 29].

Even if END is a commonly performed procedure for staging of head and neck cancer in dogs, SLNB is gaining increasing acceptance, especially in dogs with no clinical evidence of nodal metastases. This is due to high detection rates (DR) and diagnostic accuracy reported for various mapping techniques, including contrast‐enhanced ultrasound (DR 80%) [23], indirect computer tomographic lymphography (DR 55%–97%) [22, 25, 26], near‐infrared lymphography (DR 91%) [21], and lymphoscintigraphy (DR 83%) [20]. Unfortunately, there is a paucity of studies including information on surgical complications of lymphadenectomy in canine head and neck tumours. Only minor and self‐limiting complications have been reported in one study on dogs undergoing SLNB guided by radiopharmaceutical and blue dye for head and neck nodal staging, which consisted of oedema of the muzzle and seroma formation occurring in 21% of cases [20]. Although recent studies on SLNB suggest a potentially higher complication rate associated with mandibular and retropharyngeal lymphadenectomy [30, 31], complications related to END in dogs also appear to be generally self‐limiting, although their true incidence has not been clearly established [16, 25, 32]. Based on the current body of literature, it is therefore not possible to determine whether SLNB is correlated with a lower morbidity compared to END in dogs with head and neck malignancies, as happened for humans. This retrospective cohort study aims to report the cumulative incidence and severity of surgical complications related to END and SLNB in a cohort of dogs with malignant head and neck tumours, and to compare the morbidity between the two surgical approaches.

## 2 Materials and Methods

For this retrospective cohort study, the medical records of client‐owned dogs referred to two teaching hospitals (Small Animal Hospital, University of Zurich—SAH—and Veterinary Teaching Hospital, University of Milan—VTH) for surgical management of head and neck malignancies between August 2021 and August 2023 were reviewed. Inclusion criteria were:

– Absence of distant metastasis based on tumour staging performed with the current guidelines for each tumour type.
– Lymphadenectomy (END or SLNB), concurrent with curative‐intent surgical resection of a cytologically or histologically confirmed malignancy of the head and neck, or with the re‐excision of a tumour‐infiltrated scar, or alone if no surgical approach to the nodes was previously attempted in association with a complete primary tumour excision.
– Availability of detailed intraoperative and postoperative records, including the number of lymph centres surgically explored and lymph nodes excised, the type of node‐guided‐mapping technique used for SLNB, and intra−/post‐operative complications.
– Minimum follow‐up of 30 days.

Dogs were excluded if they underwent non‐guided removal of a single lymph centre (regional lymphadenectomy) or the unilateral lymph centres only.

All clinical procedures and medical or surgical interventions on dogs were performed in accordance with the available evidence at the time of the study and in full compliance with national animal welfare legislation. Informed written consent for both the surgical procedure and data collection was obtained from the owners at the time of treatment.

Retrieved variables included: signalment (breed, sex, age and body weight), clinical characteristics of the primary tumour (tumour type, first presentation vs. scar vs. recurrence, site, size, ulceration), clinical (enlarged vs. normal‐sized), imaging (normal vs. abnormal) and cytological status of the regional nodes (if present), type of surgical approach to the nodes (END vs. SLNB), number and location of excised nodes, total surgical time (primary tumour/scar excision plus END or SLNB, defined from the first incision to last suture) and surgical complications at the site of tumour/scar excision and lymphadenectomy. All samples were submitted to histopathology and tumour histotype and margins, as well as lymph nodes status, were recorded.

The approach to the nodes was categorised as follows: SLNB if a preoperative and/or a guided intraoperative SLN mapping method (lymphoscintigraphy plus gamma probe or near‐infrared fluorescence) was used to identify and remove only the SLN, without excision of the remaining non‐sentinel nodes within the surgically explored lymph centre; and END when the entire mandibular and medial retropharyngeal lymph centres were excised bilaterally, as described by Green and Boston [19]. In case the surgeons decided to perform the removal of at least one superficial cervical lymph node in addition to END, this data was also collected.

All the surgical procedures, including lymphadenectomies, were performed by a specialised surgeon (ECVS‐boarded or senior professor). Choice between END and SLNB, as well as the selection of the SLN mapping methods, was based on the availability of mapping techniques at each institution and on the clinical decision‐making of the attending surgeon made at the time of case management. Excised tumours/scars and nodes were submitted for histopathology and histotype, margin status and metastatic status of the nodes were retrieved.

Surgical complications at the tumour site or lymphadenectomy site were defined as intraoperative if they occurred between anaesthesia induction and extubation, and postoperative if they occurred thereafter. Types of surgical complications were defined based on the National Cancer Institute dictionary and VCOG‐CTCAE v2 guidelines [33]. Severity of postoperative complications was categorised according to Clavien‐Dindo (Table 1) [34]. Dogs that did not develop a postoperative complication within 30 days were considered complication‐free [35]. For statistical purposes, the complications were categorised as ‘mild’ if graded 1 or 2 of the Clavien‐Dindo classification, or ‘severe’ if graded 3, 4 or 5 (Table 1).

**TABLE 1:** Classification of surgical complications as reported by Clavien‐Dindo [34].

Grade	Definition
Grade I	Any deviation from the normal postoperative course without the need for pharmacological treatment or surgical, endoscopic and radiological interventions
	Allowed therapeutic regimens are: drugs as antiemetics, antipyretics, analgesics, diuretics, electrolytes, and physiotherapy. This grade also includes wound infections opened at the bedside.
Grade II	Requiring pharmacological treatment with drugs other than such allowed for grade I complications Blood transfusions and total parenteral nutrition are also included
Grade III	Requiring surgical, endoscopic or radiological intervention
Grade IIIa	Intervention not under general anaesthesia
Grade IIIb	Intervention under general anaesthesia
Grade IV	Life‐threatening complication (including CNS complications)^a^ requiring IC/ICU management
Grade IVa	Single organ dysfunction (including dialysis)
Grade IVb	Multiorgan dysfunction
Grade V	Death of a patient
Suffix ‘d’	If the patient suffers from a complication at the time of discharge, the suffix ‘d’ (for ‘disability’) is added to the respective grade of complication. This label indicates the need for a follow‐up to fully evaluate the complication.

Abbreviations: CNS, central nervous system; IC, intermediate care; ICU, intensive care unit.

^a^ Brain haemorrhage, ischemic stroke, subarachnoid bleeding, but excluding transient ischemic attacks.

The duration of hospitalisation was based on clinical needs. Follow‐up examinations were scheduled between 7–14 days post‐surgery and at 30 days. Additional check‐ups, either before or after the scheduled ones, were conducted upon the owner's request or in cases of complications identified during a previous examination, continuing until full resolution.

### 2.1 Statistical Analysis

Categorical variables were reported as percentages, and continuous variables as median and range. The primary outcome was the occurrence of complications at the lymphadenectomy site within 30 days. Given the presence of patients who died within 30 days without evidence of complication, possibly avoiding the observation of the occurrence of the primary outcome, a method for competing risks was adopted to estimate the cumulative incidence of complication of lymphadenectomy during follow‐up and to estimate the prognostic impact of the covariates. The regression model of Fine and Gray was applied for univariate and multivariate analysis. Categorical variables were included by dummy coding, and continuous variables in their original measurement scales. For the latter, the putative presence of a nonlinear relationship was tested, and the results of the simple model with the linear term only were reported when the contribution of nonlinear terms was not statistically significant. Model results were reported as sub‐distributional hazard ratios, 95% confidence intervals, and Wald statistic *p*‐value. Although sub‐distributional hazard ratios are complex to be clinically interpretable, they are directly linked to cumulative incidences. Thus, when a sub‐distributional hazard ratio is significantly different from 1.00, this implies a statistically significant prognostic impact of the variable on cumulative incidences [36]. Sub‐distribution hazard ratios (SDHR) did not allow quantifying the impact of the variables on cumulative incidences; hence, the model estimates of the ratio of cumulative incidences (Relative Risks, RR) at 7 and 30 days were also reported.

Given the limited number of complications, it was only possible to include some variables to obtain their adjusted estimated prognostic effect in multivariate analysis. A model with a maximum number of four variables was fitted [37]. The following variables were previously selected based on the significance of the univariate model and clinical relevance: SLN management (END vs. SLNB), number of nodes resected, clinical status of the nodes (enlarged vs. normal‐sized) and institution.

Forest plots are also provided to represent the results of the sub‐distributional hazard ratio and the relative risks of each variable considered in the models described above. That graphical representation permits the evaluation of the prognostic impact of the variables on cumulative incidences of complications more straightforwardly because it shows the estimates (SDHR and RR) and the relative confidence intervals. The coefficients are represented by filled circles in the plots. If the filled circle of the variable is to the right of the vertical line, the impact of the variable on the cumulative incidence of the complication is positive; otherwise, it is negative. In the multivariate analysis, RR was not reported since the measure cannot be referred to a single variable.

The prognostic impact is not statistically significant if the confidence intervals, represented by lines around the filled circle, intersect the value 1 (represented by a vertical dashed line in the plots).

Statistical analyses were performed using R software (version 4.4.0; R Foundation for Statistical Computing, Vienna, Austria) and the packages *cmprsk* (version 2.2‐12) and *ggplot2* (Wickham H. *ggplot2: Elegant Graphics for Data Analysis*. Springer‐Verlag, New York, 2016).

## 3 Cell‐Line Validation Statement

Not relevant to this study.

## 4 Results

Seventy‐six dogs were included in the study; 47 (61.8%) were managed at VTH (Milan) and 29 (38.2%) at SAH (Zurich) (Table S1). Median age at presentation was 9 years (range 0.5–16 years), and median weight was 24.9 kg (range 3.7–62 kg). There were 53 males (69.7%; *n* = 30 intact; *n* = 23 castrated) and 23 females (30.3%; *n* = 18 neutered; *n* = 5 intact). Mixed breed (*n* = 16; 21%) and Retrievers (*n* = 17; 22.4%) were the most common breeds. Integumentary mast cell tumour (MCT) was the most frequent tumour type (*n* = 26; 34.2%), followed by oral malignant melanoma (OMM) (*n* = 25; 32.9%), and oral squamous cell carcinoma (Oral SCC) (*n* = 7; 9.2%). Other tumour types were oral soft‐tissue sarcoma (*n* = 4; 5.3%), cutaneous SCC (*n* = 4; 5.3%), cutaneous malignant melanoma (*n* = 4; 5.3%), salivary gland carcinoma (*n* = 3; 3.9%), and osteosarcoma (*n* = 3; 3.9%) (Table S1). Forty‐five dogs (59.3%) had a first‐presentation tumour, 15 (19.7%) had a scar from previous incomplete excision, 8 dogs (10.5%) had a local recurrence and 8 (10.5%) were referred for lymphadenectomy after complete tumour excision. Excluding scar, median tumour size was 2.3 cm (range 0.2–4.5 cm). Neoadjuvant therapies were administered to 14 dogs (18.4%), all with MCT (cetirizine only or cetirizine and prednisolone, *n* = 10; chemotherapy, *n* = 2; chemotherapy plus cetirizine, *n* = 1; and radiation therapy plus chemotherapy, *n* = 1).

The RLN were judged clinically normal at palpation in 56 dogs (73.7%) and enlarged in 20 (26.3%). CT characteristics of the nodes were available in 55 cases (72.4%), and abnormalities in size or contrast uptake were reported in 20 (36.4%). In 9 cases (11.8%), lymph nodes were judged abnormal both at palpation and CT, whereas in 11 cases (14.5%), CT findings were abnormal, but palpation was unremarkable and in 4 cases (5.3%) palpation was abnormal and CT unremarkable.

SLNB was performed in 48 dogs (63.2%) (Table 2). SLBN was guided by lymphoscintigraphy and methylene blue in 26 cases (54.2%), by near‐infrared fluorescence (NIRF) in 13 cases (27%), by NIRF and lymphoscintigraphy in 4 cases (8.3%), and by lymphoscintigraphy alone in 5 cases (10.4%). In the SLNB group, a single lymph centre was identified as the sentinel and therefore surgically explored in 25 dogs, while in 23 dogs, the mapping method guided the surgical exploration of > 1 sentinel lymph centre. In the SLBN group, a median of 2.5 lymph nodes per dog (range 1–8) were removed.

**TABLE 2:** Characteristic of the population included.

Pre‐surgical variables	END (total = 28)	SLNB (total = 48)
Institution		
SAH (Zurich)	17 (61%)	12 (25%)
VTH (Milan)	11 (39%)	36 (75%)
Gender		
Female	10 (36%)	13 (27%)
Male	18 (64%)	35 (73%)
Breeds		
Mixed	9 (32%)	7 (15%)
Retrievers	5 (18%)	12 (25%)
French Bouledogues	3 (11%)	2 (4%)
Others	11 (39%)	27 (56%)
Weight		
≤ 10 kg	6 (21%)	13 (27%)
> 10 and < 30 kg	14 (50%)	16 (33%)
≥ 30 kg	8 (29%)	19 (40%)
Lymph nodes, clinically		
Normal	15 (54%)	41 (85%)
Enlarged	13 (46%)	7 (14%)
Histotype		
Mast cell tumour	10 (36%)	16 (33%)
Malignant melanoma	11 (39%)	18 (38%)
Squamous cell carcinoma	4 (14%)	7 (15%)
Osteosarcoma	1 (4%)	2 (4%)
Salivary gland carcinoma	0	3 (6%)
Soft tissue sarcoma	2 (7%)	2 (4%)
Median number of removed		
Lymph centres	4	1
Lymph nodes	6.5	2.5

Abbreviations: SAH, Small Animal Hospital, University of Zurich; VTH, Veterinary Teaching Hospital, University of Milan.

Elective neck dissection was performed in 28 dogs (36.8%) (Table 2), and in 5 of those, the superficial cervical lymph nodes were also bilaterally removed. In this group, a median of 6.5 lymph nodes per dog (range 4–8) was removed.

Overall, including dogs that received both END and SLNB, a median of 2 (range, 1–6) lymph centres were surgically explored, and a median of 3 (range, 1–8) lymph nodes were excised.

In the SLNB group, a single lymph centre was identified as the sentinel and therefore removed in 25 dogs, while in 23 dogs, the mapping method guided the removal of > 1 sentinel lymph centre.

At least one excised node was histologically metastatic in 25 dogs (32.9%), of which 10 underwent END and 15 SLNB.

Overall, the median total duration of surgery was 95.5 min (range, 23–240 min); in four cases, this data was not available. The median duration of surgery was 90 min (range, 23–205 min) in the SLNB group and 120 min (range, 35–240 min) in the END group.

**FIGURE 1:**
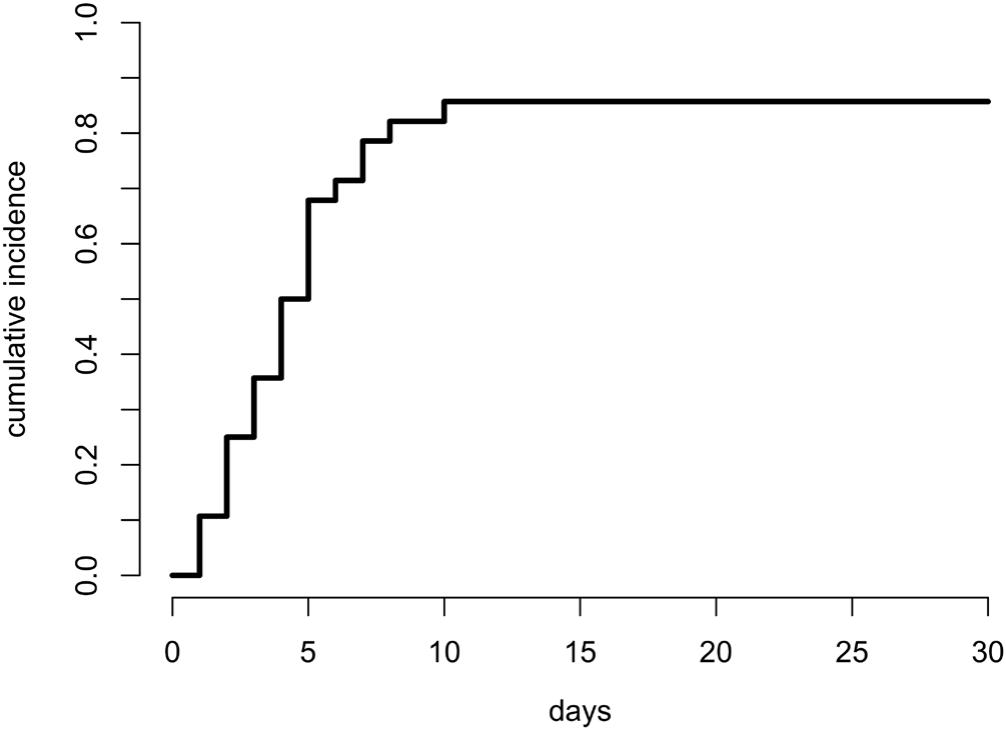
Estimated overall cumulative incidence of postoperative complications at the lymphadenectomy site.

No intraoperative surgical complications were reported. Postoperative complications occurred in 23 dogs at the tumour resection site and in 36 dogs at the lymphadenectomy site. Ten dogs experienced postoperative complications at both lymphadenectomy and tumour sites (in one case, the two sites anatomically corresponded). The cumulative incidence of complications at the lymphadenectomy site at 30 days was 47.4% (95% CI: 35.7%–58.1%) (Figure 1). Complications at the lymphadenectomy site were mild (Clavian‐Dindo grade 1–2) in 31 of cases (86%), and severe (Clavian‐Dindo grade 3–5) in 5 (14%).

Of the five dogs that experienced severe complications after lymphadenectomy, 4 (14.3%) underwent END, and 1 (2%) had SLNB. Seroma was the most common complication after lymphadenectomy, occurring overall in 32/36 (89%) dogs that experienced a complication. Type and severity of complications at the tumour site and lymphadenectomy site are detailed in Table 3.

**TABLE 3:** Postoperative complications at the tumour site and lymphadenectomy site in the study population.

	All complication No. of dogs (%)			Mild (Clavian‐Dindo Grade 1–2) No. of dogs (%)			Severe (Clavian‐Dindo Grade 3–5) No. of dogs (%)		
	Total	END	SLNB	Total	END	SLNB	Total	END	SLNB
Tumour resection site (68 out of 76 dogs, 8 dogs underwent lymphadenectomy only)									
No Complications	45	16	29	0	0	0	0	0	0
Complications	23	8	15	15	6	9	7	2	5
Dehiscence	11	3	8	7	3	4	4	0	4
Infection	4	3	1	3	2	1	1	1	0
Dehiscence + infection	3	2	1	1	1	0	2	1	1
Seroma	3	0	3	2	0	2	0	0	0
Hematoma/bleeding	1	0	1	1	0	1	0	0	0
Dehiscence + hematoma/bleeding	1	0	1	1	0	1	0	0	0
Lymphadenectomy site (76 dogs)									
No complications	40	4	36	0	0	0	0	0	0
Complications	36	24	12	31	20	11	5	4	1
Seroma	22	14^a^	8	22	14^a^	8	0	0	0
Infection	2	2	0	2	2	0	0	0	0
Dehiscence	1	0	1	0	0	0	1	0	1
Seroma + infection	4	3	1	1	0	1	3	3	0
Seroma + dehiscence	2	2	0	1	1	0	1	1	0
Seroma + edema	3	3	0	3	3	0	0	0	0
Seroma + facial nerve paralysis	1	0	1	1	0	1	0	0	0
Facial edema	1	0	1	1	0	1	0	0	0

Abbreviations: END, elective neck dissection; SLNB, sentinel lymph node biopsy.

^a^ In five cases, dogs underwent END plus superficial cervical.

When considering the lymphadenectomy sites, there were 12 postoperative complications in dogs treated with SLNB and 24 in dogs treated with END. One dog of the SLNB group died without lymphadenectomy site complications before the considered time of the study (30 days). Moreover, the cumulative incidence of postoperative complications at the lymphadenectomy site at 30 days was 25% (95% CI: 13.8%;37.9%) with SLNB and 85.7% (95% CI:64.3%; 94.8%) with END, and the difference was statistically significant (*p* < 0.001; SDHR = 6.16; RR at 30 days: 3.35). Elective neck dissection was subdivided into END and END plus superficial cervical node extirpation: there were 12 postoperative complications in SLNB, 19 in END and 5 in END plus superficial cervical. All the dogs in the group of END plus superficial cervical had a complication.

**FIGURE 2:**
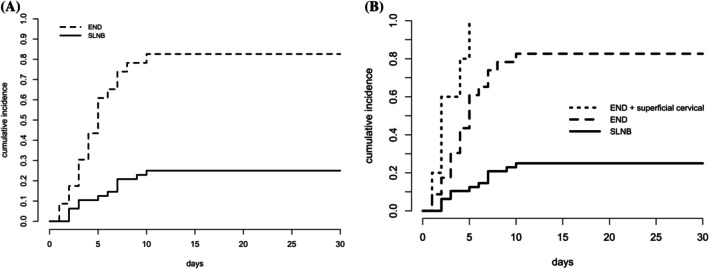
Cumulative incidence of postoperative complications at the lymphadenectomy site in dogs receiving SLNB vs. END (A), and receiving SLNB vs. END vs. END plus superficial cervical vs. SLNB (B).

When compared to SLNB, the cumulative incidence of postoperative complication differed statistically between all the three categories, with the highest recorded for END plus superficial cervical node extirpation (compared to SLNB: *p* < 0.001; SDHR = 14.8; RR at 30 days: 4), followed by END (compared to SLNB: *p* < 0.001; SDHR = 5.5; RR at 30 days: 3.2). The curves of cumulative incidence of complications for each type of cervical nodes management are shown in Figure 2.

**FIGURE 3:**
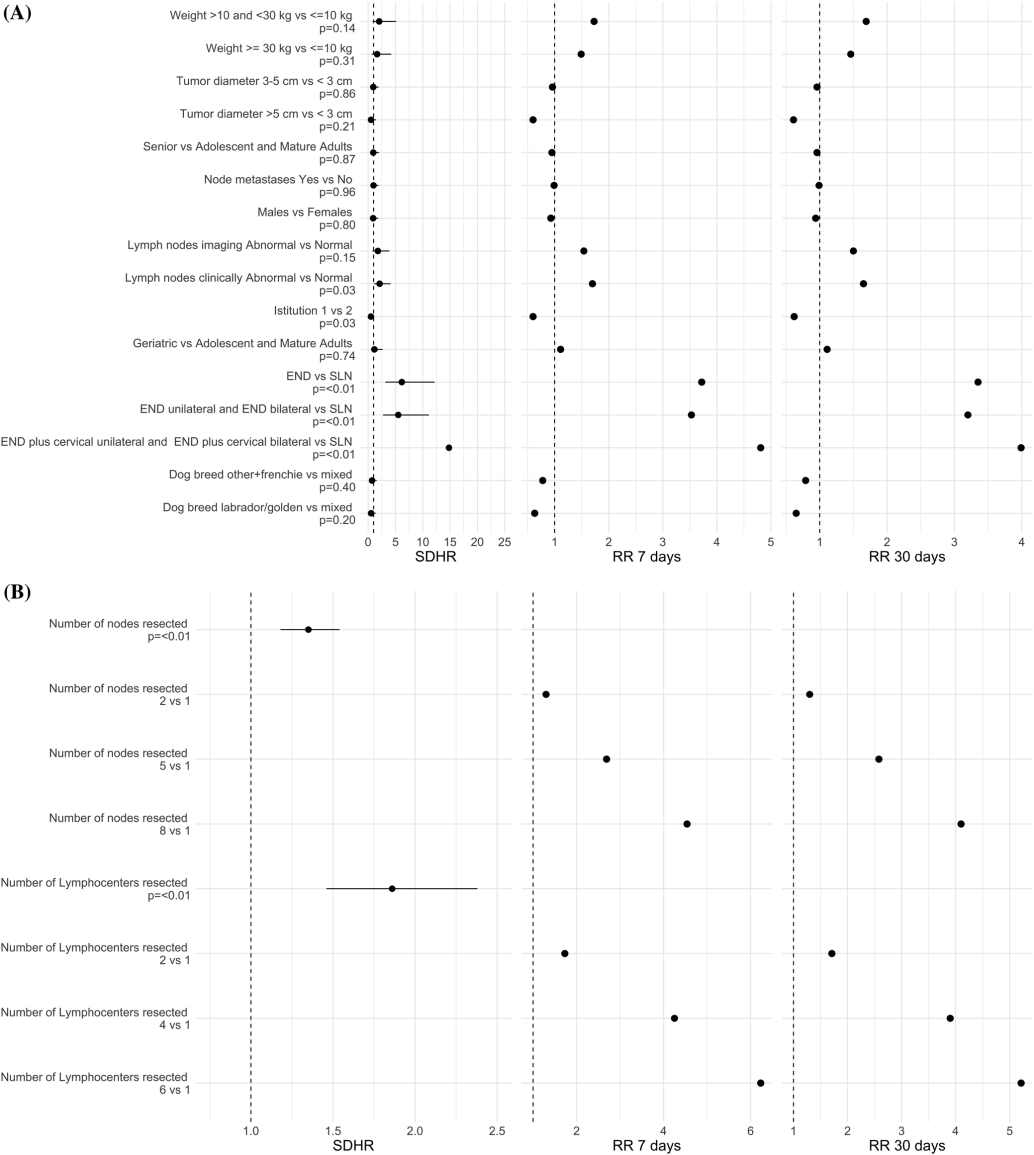
Forest plots of Fine and Gray model (univariate analysis). RR 7 days, relative risk at 7 days; RR 30 days, relative risk at 30 days; SDHR, sub‐distribution hazard ratio. Filled circles represent the model results for each variable. Lines around the filled circles represent the 95% confidence intervals. Panel A: Categorical variables—Institution 1 = Small Animal Hospital, University of Zurich, Institution 2 = Veterinary Teaching Hospital, University of Milan; Panel B: Continuous variables. In panel B, SDHR is the ratio of sub‐distribution hazard for each unit increase of the variable, and RR is the ratio of model‐estimated cumulative incidence for selected values of the variables and the cumulative incidence for the reference variable value (1.0).

In the univariate analysis, clinically enlarged RLN (*p* = 0.03; sub‐distribution hazard ratios [SDHR] = 2.1; relative risk [RR] at 30 days: 1.6), increasing number of resected lymph centres (*p* < 0.001; SDHR = 1.86 for each lymph centre) and lymph nodes (*p* < 0.001; SDHR = 1.35 for each lymph node), and institution (*p* = 0.03; SDHR = 0.5; RR at 30 days = 0.6) were significantly correlated with an increased risk of postoperative complications at lymphadenectomy site (Figure 3). Compared to the removal of a single lymph centre, the RR of developing postoperative complications at 30 days increased progressively: 1.7‐fold with the removal of 2 lymph centres, 2.7‐fold with 3, 3.9‐fold with 4, 4.8‐fold with 5, and 5.2‐fold with 6. Similarly, when considering individual lymph nodes, the RR at 30 days increased by a factor of 1.3 with the removal of 2 nodes, 1.7 with the removal of 3 nodes, 2.1 with 4 nodes, 2.5 with 5 nodes, 3.1 with 6 nodes, 3.7 with 7 nodes, and 4.1 with 8 nodes removed.

**FIGURE 4:**
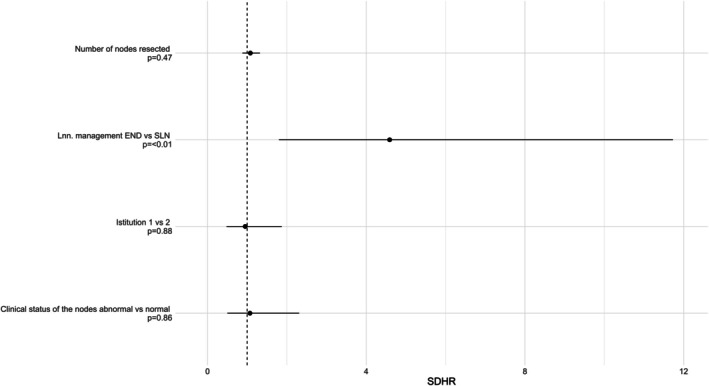
Forest plots of Fine and Gray model (multivariate analysis). SDHR, sub‐distribution hazard ratio. Filled circles represent the model results for each variable. Lines around the filled circles represent the 95% confidence intervals. For the continuous variables, SDHR is the ratio of the sub‐distribution hazard for each unit increase of the variable. Institution 1 = Small Animal Hospital, University of Zurich, Institution 2 = Veterinary Teaching Hospital, University of Milan.

In multivariate analysis, only the type of lymph node management (END vs. SLNB) was independently associated with a higher risk of postoperative surgical complications (*p* = 0.002; SDHR = 4.6), while clinical status of the nodes, number of lymph centres and lymph nodes resected, and institution lost their significance (Figure 4).

Since most of the complications were mild, the time of hospitalisation after surgery was never influenced by the postsurgical complications at the lymphadenectomy site. In the five severe complications, additional returns to clinic and medical procedure (e.g., antibiotics, surgical revision) were required, but no additional hospitalisation occurred.

## 5 Discussion

Elective neck dissection has recently gained increasing popularity for the management of dogs with various malignancies of the head and neck; however, the potential morbidity related to the procedure may be difficult to justify in dogs with a low risk of multifocal nodal metastases [16, 17, 19, 32]. The SLNB provides a more targeted approach, which can potentially spare multiple unnecessary lymphadenectomies [20, 21, 38]. The present study population compared the morbidity of these two approaches: as expected, the cumulative incidence of surgical complications at the lymphadenectomy site was significantly higher in dogs treated with END compared to SLNB. Furthermore, complications occurred more frequently when superficial cervical nodes were also included in the END procedure.

In accordance with the previous literature, in our study, most of the complications related to lymphadenectomy were self‐limiting (Clavian‐Dindo 1–2) [16, 20, 26]. The cumulative incidence of complications at 30 days after SLNB was 25%, and severe complications (Clavian‐Dindo 3–5) occurred only in 1 dog. This result is consistent with a previous study on SLNB in canine head and neck malignancies, where the authors report only self‐limiting complications with a cumulative incidence of 22% [20]. Our results also align with the complication rate of 26% reported after medial retropharyngeal lymphadenectomy by Ciammaichella et al. [31]. Similarly, surgical complications after SLNB guided by radiopharmaceutical in 113 dogs with various tumour types and locations occurred in 21.24% of cases, of which only 8.3% were severe [35]. In another recent study, a lower complication rate of 7.5% was reported in dogs undergoing superficial lymphadenectomies at various anatomical sites; however, the rate of complications for the retropharyngeal and mandibular lymph centre was respectively 40% and 11.8%, which again is consistent with the higher cumulative incidence that we recorded in our cohort of dogs [30].

Complications after END occurred in 85.7% of dogs included in the present study, and in 4 cases they were severe (Clavian‐Dindo grade 3–5). There is a paucity of data in the literature on complications of END in dogs, with conflicting results. Grimes et al. reported lymphedema and abscess formation in 6% of dogs with OMM after excision of the mandibular and retropharyngeal lymph centres [16]. Conversely, in a recent study on 39 dogs with oral tumours, most of them developed post‐END swelling, and in 13% of cases this warranted further diagnostic tests to exclude surgical site infection [26]. The high cumulative incidence of postoperative complications related to END that we report is consistent with the observations in the latter study. Although most of the complications that we recorded after END were mild (Clavian‐Dindo grade 1–2), they may still cause discomfort to the animal as well as the need for further clinical rechecks or diagnostics, which ultimately result in increased costs for the owner.

Regarding the type of complications, seroma was the most frequent finding, and this is consistent with the study by Green et al. [19], which described the surgical technique for bilateral removal of mandibular and medial retropharyngeal nodes. The authors identified seroma as a common sequela due to the creation of dead space and lymphatic disruption. The present findings also align with a recent survey study where 20% of respondents reported seroma as a complication occurring in 75%–100% of cases treated with END [32]. In the latter survey study, infection was noted as a rare occurrence by most respondents, and other less frequently reported complications included facial swelling, regional oedema, wound dehiscence, haemorrhage and muzzle oedema [32].

An increasing number of excised lymph nodes and lymph centres was also correlated with a higher cumulative incidence of complications in the univariate model. These results underscore that, although lymphadenectomy is a generally well‐tolerated procedure in dogs, a greater surgical dose results in increased morbidity. The fact that both the number of lymph nodes and lymph centres lost significance in multivariate analysis is mainly due to their correlation with the type of lymph node management. Intuitively, when END is performed, a greater number of nodes is removed compared to SLNB; hence, the statistical significance of the number of nodes and lymph centres removed is lost when the impact of the type of nodal management is also considered.

Clinically enlarged RLNs and surgery performed at the Small Animal Hospital at the University of Zurich were also correlated with a higher cumulative incidence of complications in univariate analysis, although both variables lost their significance in multivariate analysis when evaluated in conjunction with the type of lymph node management. It could be argued that in the case of enlarged nodes, a greater degree of tissue dissection is required to remove them. Another possible explanation is that dogs with enlarged nodes were more likely to be offered END than SLNB (generally proposed in clinically normal/non‐palpable nodes) [20, 39], leading to a form of selection bias that may also explain the result of multivariate analysis. Conversely, it could be argued that a non‐palpable or normal‐sized lymph node could be difficult to intraoperatively identify, possibly increasing time and tissue dissection. However, in this caseload, the use of intra‐operative‐guided mapping techniques limits this possibility for the SLNB group, even if this consideration could be true for non‐palpable or normal‐sized lymph nodes in the END group.

Regarding the impact of the institution on the cumulative incidence of complications, since a higher number of END were performed at SAH (Zurich) it seems reasonable to assume that the higher cumulative incidence of complications at this institution was due to the prevalence of the type of lymph node management. This assumption is also corroborated by the loss of significance of the variable ‘institution’ in multivariate analysis.

Limitations of the present study are mainly related to its retrospective nature. First, patients were not randomised to receive END or SLNB, but the type of nodal management was decided based on the availability of mapping methods at each institution and on the preferences of the responsible surgeon. As previously discussed, according to the results of the univariate model, cases with clinically enlarged lymph nodes were more likely to receive END than SLNB. Indeed, the presence of lymphadenomegaly may have led the surgeon to suspect a more aggressive oncologic disease, therefore prompting a more extensive surgical approach. Conversely, there was no significant difference in the distribution of tumour types between dogs receiving END and SLNB in the present study.

Another limitation of this study is that the retrospective design precluded the possibility of reliably evaluating the influence of END vs. SLNB on oncologic outcome. The effect of the removal of multiple lymph centres on survival time and disease‐free interval must be further investigated for each tumour type. This is crucial to identify those dogs that may still benefit from END despite the higher morbidity.

Finally, the low cumulative incidence of severe complications hampered the stratification based on the Clavian‐Dindo classification for statistical purposes. Future, prospective, and randomised clinical trials on a larger sample are warranted to compare the incidence of complications of various degrees of severity between the two techniques, and to statistically evaluate the non‐inferiority oncologic impact of SLNB.

In conclusion, SLNB (intraoperatively guided by NIRF or radiopharmaceutical) had a significantly lower morbidity compared to END in the present population of dogs undergoing lymphadenectomy for staging purposes of a head and neck tumour. In cases where END is planned, the result of the present study can assist the clinician in pre‐operative communication with the owners about the potential complications associated with END. Ideally, canine patients should be stratified for the risk of multifocal nodal metastases both between tumour types and within each tumour type, based on tumour grade as well as patient and tumour characteristics. This stratification would help identify those who may truly benefit from a more extensive lymph node dissection, such as an END, while sparing those at lower risk of nodal metastases from the higher morbidity associated with this procedure.

## Funding

The authors have nothing to report.

## Ethics Statement

This study is a non‐experimental clinical project based on the retrospective collection of data from dogs routinely admitted to the University Veterinary Hospitals for the presence of spontaneous tumours and treated in accordance with the available evidence at the time of the study and in full compliance with national animal welfare legislation.

## Consent

Informed written consent for both the surgical procedure and data collection was obtained from the owners at the time of treatment.

## Conflicts of Interest

The authors declare no conflicts of interest.

## Supporting information


**Table S1:** Data regarding institution, lymphadenectomy management, histotype and the site of the tumour.

## Data Availability

The data that support the findings of this study are available from the corresponding author upon reasonable request.
