# Supplementary material for: Comparison of Morbidity Between Sentinel Lymph Node Biopsy and Elective Neck Dissection in Dogs With Head and Neck Malignancies
Source: Vet Comp Oncol. 2026 Feb 17;24(2):313–23. doi: 10.1111/vco.70055 (PMC13161742; doi:10.1111/vco.70055)
Supplement: Supplementary file 1 — Table S1: Data regarding institution, lymphadenectomy management, histotype and the site of the tumour. [file VCO-24-313-s001.pdf]

Table S1. Data regarding institution, lymphadenectomy management, histotype, and the site of the tumour.

Institution	Node managment	Tumor	Site primary tumor
1	END	MCT	Maxilla left
1	END	OMM	Left tongue
1	END	Cutanopeus malignant melanoma	Nose
1	END	SCC	Left rostral maxilla
1	END	OMM	Left maxilla
1	END	OMM	Left rostral mandibula
1	END + CS	Cutaneous malignant melanoma	Nose
1	END	MCT	Neck
1	END	OMM	Left mandible
1	SLNB	OMM	Left mandibulary lip
1	END + CS	Oral SCC	Left tonsil
1	END	OMM	Left maxillary lip
1	END	MCT	Left maxillary lip
1	END	SCC	Planum nasale
1	END	MCT	Nose
1	END	MCT	Left maxillary lip
1	SLNB	SCC	Rostral mandibula
1	END	OMM	Maxilla
1	END + CS	OMM	Left maxilla
1	SLNB	MCT	Rigth maxillary lip
1	SLNB	OMM	Right maxillary lip
1	SLNB	OMM	Rostral maxilla
1	SLNB	OMM	Right manibula
1	SLNB	SCC	Planum nasale
1	SLNB	OMM	Left maxillary lip
1	SLNB	OMM	Left maxillary lip
1	SLNB	MCT	Right ear
1	SLNB	MCT	Right eye lid
1	SLNB	Cutaneous malignant melanoma	Left ear base
2	SLNB	Salivary adenocarcinoma	Right mandibular gland
2	SLNB	OMM	Cranial central mandibula
2	SLNB	Salivary adenocarcinoma	Left parotid gland
2	SLNB	OMM	Right caudal mandibula
2	SLNB	Oral fibrosarcoma	Left caudal mandibula
2	SLNB	Oral pleomorphic sarcoma	Right mandibula
2	SLNB	MCT	Conjunctiva left eye
2	SLNB	OMM	Right maxillary lip
2	SLNB	OSA	Right mandibula
2	SLNB	MCT	Right third eyelid

2	SLNB	OMM	Left lip and caudal maxillary gengiva
2	SLNB	OMM	Left mandibular lip
2	SLNB	Oral SCC	Left cranial mandibula
2	SLNB	OMM	Left mandibular lip
2	SLNB	MCT	Nose
2	SLNB	Oral SCC	Cranial maxilla
2	SLNB	MCT	Cranial neck
2	SLNB	OSA	Left mandibula
2	SLNB	OMM	Left mandibula
2	SLNB	Oral SCC	Cranial madibula
2	SLNB	MCT	Right mandibular lip
2	SLNB	MCT	Left pinna
2	SLNB	MCT	Cranial right neck
2	SLNB	MCT	Right muzzle
2	SLNB	MCT	Nose
2	SLNB	OMM	Lip
2	SLNB	Oral SCC	Oral floor
2	SLNB	Oral SCC	Right mandibula
2	SLNB	OMM	Righ mandibula
2	SLNB	MCT	Left muzzle
2	SLNB	Cutaneous malignant melanoma	Right muzzle
2	SLNB	Salivary adenocarcinoma	Right manibular gland
2	SLNB	OMM	Right maxillary lip
2	SLNB	MCT	Right eyelid
2	SLNB	MCT	Left pinna
2	SLNB	MCT	Tongue
2	END	Oral fibrosarcoma	Left mandibula
2	END	Oral OSA	Cranial hard palate
2	END	OMM	Right caudal maxilla
2	END	MCT	Right muzzle
2	END	MCT	Intermandibular skin
2	END	Oral fibrosarcoma	Left mandibular lip
2	END	OMM	Right lip and maxilla
2	END	MCT	Left eyelid
2	END	Oral SCC	Oral floor
2	ENS + CS	MCT	Right maxillary lip
2	ENS + CS	MCT	Righ maxillary lip

Legend: SLNB: sentinel lymph node biopsy; END: elective neck dissection; CS: cervical superficial node; OMM: oral malignant melanoma; MCT: mast cell tumour; OSA: osteosarcoma; SCC: squamous cell carcinoma
